# Flex-ability - A key concept to promote occupational health in everyday life beyond sick leave

**DOI:** 10.1177/10519815251317338

**Published:** 2025-02-17

**Authors:** Louise Karlsson, Lena-Karin Erlandsson, Anna Cregård, Lena Nordgren, Marie Lydell

**Affiliations:** 1 Centre for Clinical Research Sörmland, Uppsala University, Sweden; 2 School of Health & Welfare, Halmstad University, Halmstad, Sweden; 3 Dept. of Work Life and Social Welfare, University of Borås, Borås, Sweden; 4 Dept. of Public Health and Caring Science, Uppsala University, Uppsala, Sweden

**Keywords:** everyday life, health, health promotion, occupational balance, rehabilitation, stress, work

## Abstract

**Background:**

Sick leave and ill health due to stress are significant concerns today and negatively affect the individual, the organisations, and the community. High demands, multitasking, and inexplicit boundaries between different occupations contribute to an explanation. However, research shows that more qualitative studies are needed to better understand this issue and how to promote health in the working population.

**Objective:**

The study aims to explore participants’ experiences of maintaining or regaining occupational health in their everyday life, including paid work, several years after sick leave.

**Method:**

Nine semi-structured interviews were conducted and analysed using inductive content analysis. The analysis resulted in one major theme and three categories describing the current experiences handling their everyday life and work situations.

**Results:**

The result showed that returning to work and maintaining occupational health after sick leave due to occupational ill health calls for “flex-ability”. The term describes that individuals need to be open to change and adapt to new challenges at work and in everyday life.

**Conclusion:**

The findings highlight the importance of health-promotive organisations where the individual factor is more considered. Furthermore, a broader view of health-promotive work in society where work is included in everyday life, instead of divided into and outside of work, is needed.

## Introduction

Paid work is a crucial part of everyday life, and its impact on the perception of occupational balance is known to influence both health and ill health.^1,2^ For the individual, occupations related to paid work have a multidimensional value, both personally and socioculturally.^3^ In Sweden, there are about 200,000 individuals on sick leave, where approximately 25% are caused by stress-related ill health. The majority of those on sick leave are women within the healthcare and childcare sectors, while areas such as IT have the lowest sick leave rates.^4^ A similar pattern is seen in other countries in Europe.^5^ In contemporary society, there is an increasing acceptance and support for high levels of stress, busy schedules, and extended work hours as normative aspects of life. According to Black and Britt,^6^ it is crucial to comprehend how individuals may come to perceive elevated stressors as commendable and to understand the complex relationships that may exist in relation to performance, health, and well-being outcomes.^6^ The concept of health is similarly complex, with healthcare often dichotomising it as either well or ill. This is in contrast to the salutogenic perspective, which views health as a dynamic state that can fluctuate over time without the presence of disease.^7^

Occupation most often refers to paid work or a profession. In this study, however, the word occupation means an individual doing something in an environment in any daily area, such as paid work, schooling, household chores, rest, or leisure.^3^

In addition to sleep, paid work is the area of occupations where adults spend most of their time, having the strongest influence on their daily routines.^1,8^ Furthermore, the term occupational balance is both theoretically and empirically confirmed. Occupational balance is associated with perceived health and well-being^9^ and ability to work.^10,11^ It is construed as the extent to which an individual's unique pattern of daily occupations, considered within their specific context, supports their essential needs for well-being and quality of life.^3,12^ Our daily occupations form recurring patterns over time, within each single day,^13^ and evolve throughout our lives.^12,14^ Occupational balance is thus the perceived balance between all occupations in our daily lives throughout our lifespan.^3,9,12^

Today there are several different work rehabilitation interventions (WRI) aiming to regain or improve workability. In a review regarding the concept of workability, it was defined as “…the capacity of an individual to meet the physical, psychological, and social demands of their job…”.^15^ This concept encompasses several dimensions, such as health, skills, motivation, and work conditions, and is closely tied to the worker's ability to continue functioning effectively *at work*.^15^ The WRI, named “Redesigning Daily Occupations” (ReDO^®^),^16,17^ is based on the ValMO theory^18^ and research about patterns of daily occupations and health. The group intervention aims to support individuals perciving occupational ill health through tools and strategies in order to create and maintain occupational balance and health with time for paid work.^19^ However, unlike many other WRI, ReDO^®^ also focuses on everyday occupations outside of the job area. The intervention has shown positive outcomes regarding better health and return to work.^16,20–23^

A quantitative systematic review regarding long-term effects after participating in different types of WRI showed varied effects^24^ and the authors conclude that it is important to gain knowledge regarding which factors contribute to long-term success to promote occupational health and prevent further sick leave. Another review considering stress and WRI concluded that more qualitative studies are needed to gain a more comprehensive understanding of the process, both during and after participating in a WRI.^25^

To conclude, our study may contribute to the current knowledge base with a deepened perspective and understanding of how occupations and their interactions influence everyday life after sick leave. With a health-promotive perspective, based on a salutogenic approach, knowledge concerning which tools have worked to maintain changes in work and everyday life is urgent to promote sustainable health in everyday life and prevent individuals from needing sick leave due to occupational ill health and stress. The same understanding may prevent individuals from being at risk of returning to sick leave since they were unable to incorporate necessary and new strategies into their lives or access the proper support to enable them.

The current study departs from the experiences of individuals who participated in the WRI ReDO^®^ between 2018–2021 and have returned to work after sick leave, part- or full-time. The study aims to describe a sample of former ReDO^®^ participants’ experiences of maintaining or regaining occupational health in everyday life, as well as returning to and remaining in work.

## Materials and methods

The study used a descriptive qualitative research design with an inductive approach. An explorative qualitative methodology offers a nuanced exploration of the process of returning to work after sick leave due to occupational ill health by delving into individual experiences, emotions, and contextual intricacies. This approach is particularly crucial as quantitative studies tend to dominate current research, potentially overshadowing the intricate personal narratives and contextual nuances that qualitative methods are better equipped to capture.^26^

### Data collection and participants

The data collection was conducted in Sweden from December 2022 through to March 2023. The participants were, however, recruited within a larger project.^27^ Approximately 300 occupational therapists (OTs) trained in WRI ReDO^®^ and listed in the Swedish Association of OTs, were contacted by email, and asked to assist with the withdrawal of data from medical records. With the help of 12 OTs, a total of 276 potential participants were then contacted through mail with information about the project^27^ with a consent form included. Fifty-four individuals returned their consent to participate in the first study; 24 consented in writing to participate in the current study as well. Eighteen individuals had submitted their contact information, and through purposeful sampling (24), a total of 12 participants were considered suitable for the study, i.e., they had participated in a ReDO^®^ intervention between 2018 and 2021 and worked within one of the three sectors: healthcare, childcare/school, or IT. Since sick leave rates are highest within the health and childcare sector, and the IT sector has the lowest number,^4^ participants from these sectors were recruited from different parts of Sweden.

The participants were contacted by telephone or email between the 14^th^ of November and the 16^th^ of December 2022. Two individuals did not answer, but 10 individuals agreed to participate. Three reminders were sent out. One participant did not show up for the interview, nor did he/she answer via phone or email. Eventually, nine individuals were included. All interviews started with the participants consenting verbally to participate to complement the written consent, and where informed of their right to withdraw their consent without further questions; this was also recorded. The participants’ ages varied between 37 and 56. Seven of the participants were women (see Table 1).

**Table 1.** Sociodemographic characteristics of the participants (n = 9).

Sociodemographic factors		N
Age (min-max)	37–56	9
	W = 40–56	7
	M = 37–42	2
Children	No children/no children living at home	2
	Children living at home	7
Occupational health	Not on sick leave	4
	On sick leave 25–50%	4
	On sick leave 50–75%	1
Area of work	Healthcare	3
	Childcare/School	4
	IT/Administrative	2
Having opportunities for remote workplace part-time	Yes	5

Data was generated through semi-structured, digital interviews, enabling us to recruit from all over Sweden. Eight interviews were conducted through video meetings and one by telephone without a camera during January-February 2023. Recruiting participants, taking contacts, and performing the interviews were conducted by the first author.

An interview guide was developed which included core questions in relation to the aim of the study, including room for follow-up questions. Questions used included “What has worked well in your return-to-work situation?” regarding their occupational health with follow-ups like “Can you please develop?” or “Is there anything that aggravated it?” The interviews lasted between 38 and 6 min, averaging 50 min. During the interviews, the interviewer made notes of reflections, which were later discussed with the research team and considered in the analyses.

### Intervention

The ReDO^®^ intervention is an occupational therapy group intervention focusing on changes in everyday life.^19^ The intervention program is based on research on the connection between women's daily occupations and their health.^16,19,20,28^ Today, the programme is, however, both gender and diagnosis-neutral and directs individuals who are experiencing a need to make changes in everyday life to gain health and workability. Thus, ReDO^®^ addresses the whole occupational pattern, including paid work.^19^ Several studies have confirmed the effects of the ReDO^®^ programme.^20,22,29^

### Data analysis

The interviews were analysed using inductive qualitative content analysis.^30^ The first author carried through and transcribed the nine interviews, a total of 249 pages. The data was analysed by the first and last author. Meaning units from selected texts that related to the objective of the study were identified and extracted. All meaning units were sorted based on their content and were then condensed and abstracted into codes. This was done by putting post-it notes on a board and digitally through tables. Similar codes were grouped into categories describing the content, and an overall theme was created that described all the different categories (see Table 2). The last author was included in every step and was given all the material as the analysis transpired, and together through reflection and discussion the different categories and the overall theme were set. The coding and tentative categories were discussed mainly by the first and last authors. All authors had access to the material through a secure digital platform and their input during the process was considered in meetings and by comments in the material.

**Table 2.** Example of the analytical process in the study, moving from text to overall theme.

Meaning unit	Code	Category	Overall theme
“*I have a lot of freedom in my work situation which I am very grateful for in my job and which I like. I can control it*”	Less demands and responsibilities to cope	Workplace relationship dynamics	Flex-ability in everyday life
“*We had a support person within the municipality. She was a hugely important person for both my family and for me… She made sure, among other things, that we got some extra time at preschool so I could get some time to work out for an hour after work on Fridays before I picked up the children*”	Valuable relationships	Support from external networks	Flex-ability in everyday life
“*I've let go of the demands and that everything must be perfect… I'm more relaxed in my everyday life… Just take three deep breaths and you're grounded again somehow. Just don't take life so seriously anymore*”	Consider it a lesson learned	Personal insights and transformations	Flex-ability in everyday life

### Ethics

Ethical approval for the study was granted by the Swedish Ethical Review Board (dnr 2021-01071). All informants received a letter with a written explanation about the purpose of the study, the meaning of contribution, and that their anonymised concluding notes would be used for analysis on a group level. Information about confidentiality, free participation, and an assurance that the participants could request to withdraw their documents from the study at any time was included in the letter. Written and oral informed consent was then obtained from the participants.

## Results

**Figure 1.**
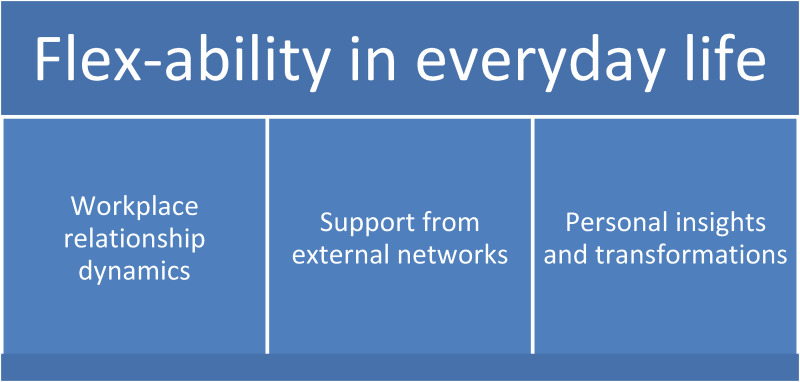
Results of the qualitative analysis regarding participants’ experiences of managing their everyday life and work situation after sick leave, showing the overall theme with three main categories.

The qualitative content analysis resulted in three categories that capture the participants’ experiences of what they used or valued in the process to maintain or regain occupational health in everyday life, two to five years after they participated in a WRI, including paid work (Figure 1). The emerging categories were *Workplace relationship dynamics, Support from external networks,* and *Personal insights and transformations.* Based on the content in the categories, one overall theme was created: “*Flex-ability in everyday life*”. Citations from the interviews are used to illustrate the results, and anonymous codes are used to protect the participants’ identities.

### Flex-ability in everyday life

The overall theme is a quibble, combining flex- (as in flexibility) and -ability. It intends to illustrate the participants’ collective experiences of trying to find their way based on their own needs and conditions, in relation to their everyday life and contexts. The participants described that the ability to adapt to change over time, whether they be external circumstances or internal shifts in their lives, was a crucial prerequisite for maintaining and promoting their occupational health. The participants described an ongoing journey with different choices to make, and challenges to handle, in relation to both their own recovery and all their occupations such as work, leisure, and home-related demands. The theme summarises a central part of the three categories exploring the participantś experiences of their everyday life today and describes their journey and changes they made to promote their occupational health after they returned to work.

The overall analysis revealed how an openness for “flex-ability”, various internal and external demands, relationships, and insights had influenced the participants’ occupational patterns in everyday life, such as how they valued different things and how they re-prioritised their occupations and relationships. The different categories illustrate how “flex-ability” related to work, relationships, and new insights could be both an asset as well as a hassle if the participants were not met with the same “flex-ability” or had a hard time adapting to changes themselves.

### Workplace relationship dynamics

In this category, the “flex-ability” was illustrated by the participants’ descriptions of interactions and dynamics in workplace relationships, both with the participants’ employers and colleagues. This aspect acknowledges the significance of work-related relationships in the broader pursuit of occupational balance.

The relationships were described by the participants as both a resource in promoting their health by giving support and feedback, and as something that could be hard to manage and navigate. To find and pilot new ways of being and communicating based on their needs, not going back to the way it was before the sick leave, showed to be a struggle. Another struggle was to get the right support at the right time, to find a sustainable way to be back at work. Some participants experienced the support from their employer and the boundaries that were set as key, while others struggled with it due to the perception of being seen as less competent due to the reduced workload. The participants expressed the pivotal role of supportive employers, describing instances where understanding managers facilitated a smoother return to work, alleviating stress and helping them navigate work tasks.

“It was incredibly frustrating not being able to get started, so it was tough to take it slow… But she (the manager) just didn't want to push me too fast” (9, p7)

The support from the employer was described as central and important in relation to the participants’ occupational health. The support included having someone who saw you, helped you to prioritise at work, and made sure that the work tasks were suitable and meaningful. It also included the possibility to spend time with colleagues and creating and supporting good relationships, including communication, collaboration and sometimes even friendships outside of work. The participants also described the importance of having that “individual” factor considered at work in relation to their occupational health, i.e., their personal needs were considered. When the participants were met with “flex-ability”, for example, by their employer enabling various working hours or other tasks, it facilitated their occupational balance and health. For example, participants appreciated being able to work from home a few days a week. The participants also mentioned the importance of individual considerations in their work situation as key to feeling in control and to reduce stress in everyday life.

“I have a lot of freedom in my work situation which I am very grateful for in my job and which I like. I can control it” (5, p6)

However, for some participants “flex-ability” could not be offered in their contexts, which seemed to negatively impact their everyday life and occupational health.

“I've sort of asked if there are other things I can do. I have understood that no, I cannot do anything from home. I have understood that. But I have asked for more administrative tasks, for example, because I think I am very good at writing and formulating me in writing and such. But my manager just says that that's not possible…” (2,p11)

The participants highlighted the importance of positive workplace relationships, emphasising the supportive nature of their colleagues as impacting on their overall occupational health. To have good relationships in the workplace, where you can feel safe, dare to ask for help, and laugh together was described as a key factor for the participants’ occupational health, both in the return-to-work process and in their work life today.

“We have great fun together, we are a nice work group and there is a lot of support for each other, through thick and thin. Yes, we are like a small family” (1, p7)

### Support from external networks

In this category the experiences of “flex-ability” regarding relationships in the participants’ everyday life, outside work, were described as an important factor for finding and maintaining a balanced rhythm in everyday life and occupational health. Both positive and negative aspects of relationships were depicted.

In the process after the rehabilitation, the participants described how valuable it was to get the right support at the right time, to enable room for recovery and meaningful occupations. Sometimes the support and “flex-ability” came from friends and family, but also from people in organisations such as the social insurance agency or the municipality.

“We had a support person within the municipality. She was a hugely important person for both my family and for me… She made sure, among other things, that we got some extra time at preschool so I could get some time to work out for an hour after work on Fridays before I picked up the children” (8, p7)

Furthermore, having close and meaningful relationships to get support and validation in everyday life could help with navigating and creating balance. For those who lived with a partner, shared responsibility for the kids and the chores at home was seen as a positive factor.

“My husband and I are a good team too… it works very well… We simply have a steady life” (6, p12)

The participants described that some people in their context did not really consider their state of well-being and were unable to give them the right type of support. Instead of listening and supporting, they made them feel bad, lazy, or contributed to feelings of stress and ill health. Instead of giving a helping hand, negative comments were made that made them feel guilty.

“My mother-in-law doesn’t always understand. She tells me what needs to be done around the house and I’m thinking yes I know but… then my mother comes over for coffee and she says like, well here it's still Christmas and it's February (2, p19)

The participants described that they had started to list their relationships in everyday life and realised that some relationships drained them of energy rather than fuelled them. These relationships took both time and energy which contributed to their sense of occupational ill health and led to an imbalance in everyday life. To maintain occupational health and a sense of balance in everyday life, the participants needed to reconsider and cut some relationships and social life to make room for more meaningful occupations.

“All people do not fill me, some drain me more than they give and then you kind of have to cut it…” (9, p16)

### Personal insights and transformations

In this category the more personal aspect and insights were of importance. The participants’ re-evaluation of a more personal “flex-ability” regarding self-prioritisation and the re-prioritisation of mindsets and values were described. This category highlighted that most of the participants described themselves as “yes-sayers” before their sick leave, both at work, and among friends and family, e.g., in the context of their kids’ recreational activities. They described a transformative shift in prioritising themselves, recognising a need to balance self-care with their roles for a more sustainable and healthier lifestyle.

“I've always kind of been there for everyone else… this was like an awakening that now I must think about myself …” (1, p12)

The participants described a process of revaluing the meaning of life, that it is not just about performance and doing everything for everyone else but to start enjoying life and not take everything seriously all the time. The experience of being on sick leave made them re-evaluate how they planned and used their time and energy in a more balanced way to promote their health.

“I've let go of the demands and that everything must be perfect… I'm more relaxed in my everyday life… Just take three deep breaths and you're grounded again somehow. Just don't take life so seriously anymore” (7, p13)

However, this showed to be an ongoing process, and some participants had to go back on sick leave more than once to really understand that they had to make a transformation and find new ways of both being and living. To return to old habits and believe that everything will work out once they are back at work without making any changes only led them back to the same state of occupational ill health. The participants also described that they needed to be open to the concept of relearning, re-prioritising and questioning their mindset and values, for example, re-evaluating who they were doing things for and why.

“Practising that it's allowed to be unclean, practising leaving the laundry - it doesn't matter. Practising lowering the demands… I try to follow what the body says, instead of what the brain tries to tell me” (9, p15)

The participants described that they struggled to manage everyday life with all their different roles, but having the right job meant that work could be a source of validation where they felt competent and appreciated. This contributed to their state of well-being and self-esteem, as well as to their experience of balance, i.e., work was a health asset.

“I have a hard time with the parental role, which can be very difficult, but I am usually happy when I work! It's a big part of my identity and it gives me a sense of relief to” (8, p12)

Some participants described that they had gained new insight regarding their profession, since work was such a contributing factor to their stress levels and need to be sick listed. The participants concluded that it might not have been the right job for them at the time leading them to find new opportunities. In some cases, this led to a career switch, despite having studied for several years.

“During my last sick leave, I came to a realisation. Whilst I have a background in teaching, I felt the need to pursue a different path. I started to study media and communication science” (3, p8)

## Discussion

This study explored the experiences of former WRI participants after they had returned to work, to gain a deeper understanding of their everyday life, work situation and overall occupational health. The study findings indicate that the ability to adapt and change, i.e., to embrace change, seek opportunities for personal development and maintain an adaptive and health-promotive approach in everyday life were essential to achieving and maintaining occupational health. This “flex-ability” was found to be central in striving towards balance and occupational health in everyday life.

Most research in this field mainly focuses on either work ability or health, and a systematic review concluded that more focus needs to be on the individual's subjective well-being after a WRI.^31^ Therefore, we applied a health-promotive perspective, using a salutogenic perspective,^7^ wanting to capture the participants’ experiences of what changes had been beneficial and central in their everyday life since their participation in the WRI.

In the category “workplace relationship dynamics”, the need to feel validated, safe, and supported based upon individual needs both by the employer and colleagues showed to be key health-promotive factors contributing to occupational health. This result is in line with previous research focusing on health-promoting factors at work.^32,33^ In one study various important promotive factors were discussed with employees. These factors encompassed being needed and to receive support and tolerance adapted for each employee.^34^ For some participants, collegial cohesion was deemed important for their health and work motivation, as well as having time and opportunity for recovery. This is supported by previous research.^35^ The results indicated that enabling and encouraging such collegial exchanges often fell under the responsibility of the employer to ensure their validity, in addition to providing timely and appropriate support while possessing the necessary knowledge to do so. This aligns with findings from other research.^36^

Furthermore, to be able to receive or accept support from the employer when returning to work sometimes showed to be a struggle. This stemmed from a fear of appearing less competent or weak if they needed to change their work style or approach. However, the result showed that by being offered “flex-ability” from the employer such as being able to impact work hours, work settings, work tasks or being offered remote work opportunities could enhance the sense of control, occupational health, and balance in everyday life. To have control over the work situation and to be able to work remotely has in previous research been highlighted as health-promoting.^37^ However, there is also research showing the opposite, that remote work can be a struggle to manage since you are always “connected”, even outside of working hours^38^ and that high cognitive demands may jeopardise detachment from work.^36^ Remote work therefore seem to have the potential to promote health, but also presents risks for adverse health outcomes. These outcomes may be influenced by the individual's perception of their work tasks’ comprehensibility, manageability, and meaningfulness.^39^ To ensure that remote work fosters well-being, it is essential to provide clear organizational structures and adequate support systems.

The result highlighted the impact the employer and colleagues had on the participantś occupational health, since work is one of our main occupations in everyday life.^1,8,11^ Overall, this result illustrates how complex this subject is, with no clear guidelines to follow since the individual aspect seems to play a significant role. Different organisations also have different resources to be available for their employees, and it seems important to find the right “flex-ability” based on the conditions at each place of work. One way could be through continuous communication to create consensus on a mutual “flex-ability” balancing what the employee needs versus what the employer can offer.

In the category “support from external networks” the importance of receiving support and understanding from the participants’ surroundings was described, which is supported in previous research.^40,41^ However, the result also showed that these interactions could be a struggle in relation to occupational health. According to the ValMO theory^14^ the experience of performing an occupation can be operationalized in three supplementary occupational value dimensions; concrete-, self-rewarding-, and socio-symbolic occupational value. The socio-symbolic ocupational value, is about one's sense of identity and competence e.g., professional skills and roles in various settings, including the workplace. However, the same socio-symbolic value that gives meaning to one's daily occupations can also become a source of distress when for example expectations from collegaus or employer related to work are challenging to fulfill.

The results illustrated how hard it can be to navigate, communicate and balance needs for a better occupational health in relation to the social contexts. The result also illustrated that to have the right support with “flex-ability” was important in the rehabilitation process as well as in the present to promote their occupational health. One study exploring the social insurers’ role in the rehabilitation process showed that by adopting a customer-oriented approach, the outcome of the occupational rehabilitation as well as the health of the individual was improved.^42^ Our result showed that the support could include both physical and psychological, and be given from friends and family, but also from external sources such as the social insurance agency or municipality which also has been shown in previous research.^27^ Furthermore, it has been shown that interpersonal relationships, both in and outside of work, can be considered a valuable resource for managing stress.^43^ Overall, this result highlights the impact, both positive and negative, that others could have on the participant's occupational health. Therefore, it seems important to create a greater understanding in society and different organisations, of the impact their actions might have on others’ occupational health, but also for individuals to become more aware of their relationships and how they affect their health.

The category “personal insights and transformations” revealed how new realisations had been made, such as realising how society, norms, and values of what is “right” or “expected” had affected health in a negative way. Previously, it has been noted that as cultural norms increasingly accept high stress levels and long working hours, elevated stress levels may be perceived as honourable.^6^ Some participants had come to the realisation that they had the wrong job, while others had realised how important their job was for them in several ways. To feel that work is important and that one is good at it, has been perceived in earlier research^44,45^ as health-promoting and a self-esteem booster. The results revealed that participants who consistently prioritised others’ needs over their own struggled to regain or maintain their occupational health.

After participation in the WRI and returning to work, the participants perceived they had a deeper understanding of themselves, what they liked and which occupations promoted their health, and finding balance between them all was described as key. Gaining insight into evaluating themselves, their time and energy, and to spend it more wisely, rather than focusing on trying to be “perfect” in the eyes of others, seemed to be a crucial part in guiding the promotion of occupational balance and health. This is in line with other studies where to strive for, or to have balance in different parts of everyday life, has been found to be an important health-promoting factor.^18,27,44,46^ This result shows the importance of understanding your own needs and valuing them, and not only trying to perform what is “expected” by others.

The overall findings emphasise the importance of workplace “flex-ability” in several key areas which seemed crucial for regaining or maintaining occupational health. The results revealed that negative experiences, such as being on sick leave due to stress or occupational ill health, could over time give the participants a wake-up call they were thankful for. It made them stop, reflect, get to know themselves better and they started living their life for themselves instead of for everyone else. To be aware of one's motivation, lifestyle and capacity together with the impact of the physical and social environment could affect the ability to make health-promotive choices thus, its central for the opportunity for occupation and participation.^3^ However, the results also identified an ongoing process, and that a “flex-ability” seemed to be needed in everyday life to strive for balance and promote the participants’ long-term occupational health.

Furthermore, to promote health, it seems important for employers to have a comprehensive understanding of how to lead with a health-promotive focus and to recognise their employees’ individual overall everyday life and roles. The result of this study indicates the importance of gaining a deeper understanding of the process followed by individuals to regain or maintain occupational health in everyday life after sick leave, since everyday life and work life are highly intertwined.

### Limitations of the present study and suggestions for future research

The main strength of this study was the qualitative approach, which allowed for a deeper understanding within the research area. The interview guide was tested beforehand, and even though the material was rich, a few more interviews would have been desirable to increase the credibility of the study. The use of digital semi-structured interviews provided the participants with the possibility to elaborate and describe their journey and everyday life from their own environment of choice.

The participants were selected purposively to ensure variety and representation from the three work sectors, thus strengthening the transferability. The goal was to recruit four participants from each sector with a 50/50 split in gender, which would have included 12 participants. However, the nine interviews undertaken were very rich in material and participants of both gender and from all three sectors were recruited. To further strengthen dependability, all interviews began with the same question and consistent follow-up questions, and the participants were encouraged to talk freely and elaborate on their experiences.^30^ Overall, the material was assessed as sufficient.

A qualitative content analysis was used to gain further understanding and description of the participants’ experiences. The study aimed to stay close to the actual text of the final notes, applying an inductive, data driven content analysis.^30,47^ The main author (LK) systematically analysed the data, discussed it back and forth with ML, and had ongoing reconciliation meetings with all authors that also had full access to the transcribed material to reach a consensus. This process was intended to strengthen dependability and confirmability^48,49^ and limit possible bias due to the human factor and thus increasing confirmability.^49,50^ Nevertheless, the researchers’ previous theoretical and clinical knowledge cannot be completely disregarded, but the team of five authors coming from different scientific and empirical backgrounds ensures a broad perspective of both analysis and discussion. The confirmability of the analysis was strengthened by using quotations in linking the data to the main concepts.^51^ Although the quotations illustrated the participants’ experiences, the analysis was close to the text. This strengthens the dependability.

## Conclusion

To return and stay in work after sick leave due to occupational ill health calls for “flex-ability”. The result highlights the importance of health-promotive organisations where the individual factor is considered, both in the rehabilitation process and in everyday life. Thus, the findings call for a broader view of health-promotive work in the society where work is included in everyday life, instead of divided into work and outside work.

## References

[1] Sandqvist J Ekbladh E . Applying the model of human occupation to vocational rehabilitation. In: Kielhofner’s model of human occupation : theory and application [Internet]. 5th ed. Philadelphia: Wolters Kluwer, 2017, pp. 377–396.

[2] Hammarström A Lundman B Norberg A . The importance of having a paid job. Gendered experiences of health and ill-health in daily life among middle-aged women and men. BMC Pub Health 2021; 21: 1–11. 34742259 10.1186/s12889-021-12034-7 PMC8572442

[3] Taylor RR . Kielhofner's model of human occupation: theory and application. 5th ed. Philadelphia: Wolters Kluwer, 2017.

[4] Swedishsocialinsurenceagency. Short Analysis 2021: 3. [Report in Swedish]. 2021

[5] Swedishsocialinsuranceagency. Short Analysis 2022:4. [Report in Swedish]. 2022

[6] Black KJ Britt TW . Stress as a badge of honour: relationships with performance, health, and well-being. Work Stress 2023; 37: 222–247.

[7] Antonovsky A . The salutogenic model as a theory to guide health promotion. Health Promot Int 1996; 11: 11–18.

[8] Sandqvist J Ekbladh E . Applying the model of human occupation to vocational rehabilitation. 2017.

[9] Wagman P Håkansson C Björklund A . Occupational balance as used in occupational therapy: a concept analysis. Scand J Occup Ther 2012; 19: 322–327. 21780985 10.3109/11038128.2011.596219

[10] Wilcock AA Hocking C . An occupational perspective of health. 3 ed. New York, NY: Slack Incorporated, 2015.

[11] Schell BAB Gillen G . Willard and Spackman's occupational therapy. Philadelphia: Wolters Kluwer, 2019.

[12] Matuska KM Christiansen CH . A proposed model of lifestyle balance. J Occup Sci 2008; 15: 9–19.

[13] Eklund L-KE Mona E . Describing patterns of daily occupations-a methodological study comparing data from four different methods. Scand J Occup Ther 2001; 8: 31–39.

[14] Persson D Erlandsson L-K Eklund M , et al. Value dimensions, meaning, and complexity in human occupation-a tentative structure for analysis. Scand J Occup Ther 2001; 8: 7–18.

[15] Cadiz DM Brady G Rineer JR , et al. A review and synthesis of the work ability literature. Work Aging Retir 2018; 5: 114–138.

[16] Eklund M Erlandsson L-K . Return to work outcomes of the redesigning daily occupations (ReDO) program for women with stress-related disorders—A comparative study. Women Health 2011; 51: 676–692. 22082247 10.1080/03630242.2011.618215

[17] Eklund M Wastberg BA Erlandsson LK . Work outcomes and their predictors in the redesigning daily occupations (ReDO) rehabilitation programme for women with stress-related disorders. Aust Occup Ther J 2013; 60: 85–92. 23551001 10.1111/1440-1630.12019

[18] Erlandsson L-K Persson D . The ValMO model: occupational therapy for a healthy life by doing. 2020.

[19] Erlandsson L-K . The Redesigning Daily Occupations (ReDO)-program: supporting women with stress-related disorders to return to work—knowledge base, structure, and content. Occup Ther Mental Health 2013; 29: 85–101.

[20] Eklund M Erlandsson LK . Women's perceptions of everyday occupations: outcomes of the redesigning daily occupations (ReDO) programme. Scand J Occup Ther 2014; 21: 359–367. 24878142 10.3109/11038128.2014.922611

[21] Wastberg BA Erlandsson LK Eklund M . Women's perceived work environment after stress-related rehabilitation: experiences from the ReDO project. Disabil Rehabil 2016; 38: 528–534. 26171915 10.3109/09638288.2015.1046567

[22] Olsson A Erlandsson LK Hakansson C . The occupation-based intervention REDO-10: long-term impact on work ability for women at risk for or on sick leave. Scand J Occup Ther 2020; 27: 47–55. 31099284 10.1080/11038128.2019.1614215

[23] Fox J Erlandsson LK Shiel A . A feasibility study of the Redesigning Daily Occupations (ReDO(TM)-10) programme in an Irish context. Scand J Occup Ther 2022; 5: 415–429. 10.1080/11038128.2021.1882561 33556290

[24] Tingulstad A Meneses-Echavez J Evensen LH , et al. Effectiveness of work-related interventions for return to work in people on sick leave: a systematic review and meta-analysis of randomized controlled trials. Syst Rev 2022; 11: 192. 36064472 10.1186/s13643-022-02055-7 PMC9446672

[25] Lambreghts C Vandenbroeck S Goorts K , et al. Return-to-work interventions for sick-listed employees with burnout: a systematic review. Occup Env Med 2023; 80: 538–544. 37500536 10.1136/oemed-2023-108867 PMC10447379

[26] Franche R-L Cullen K Clarke J , et al. Workplace-based return-to-work interventions: a systematic review of the quantitative literature. J Occup Rehabil 2005; 15: 607–631. 16254759 10.1007/s10926-005-8038-8

[27] Karlsson L Erlandsson L-K Cregård A , et al. Taking control of one’s everyday life-a qualitative study of experiences described by participants in an occupational intervention. BMC Pub Health 2023; 23: 1–12. 36997894 10.1186/s12889-023-15515-z PMC10064529

[28] Eklund M Erlandsson LK . Quality of life and client satisfaction as outcomes of the Redesigning Daily Occupations (ReDO) programme for women with stress-related disorders: a comparative study. Work 2013; 46: 51–58. 23324689 10.3233/WOR-121524

[29] Eklund M Erlandsson LK Wastberg BA . A longitudinal study of the working relationship and return to work: perceptions by clients and occupational therapists in primary health care. BMC Fam Pract 2015; 16: 46. 25887461 10.1186/s12875-015-0258-1 PMC4397877

[30] Graneheim UH Lundman B . Qualitative content analysis in nursing research: concepts, procedures and measures to achieve trustworthiness. Nurse Ed Today 2004; 24: 105–112. 10.1016/j.nedt.2003.10.001 14769454

[31] Figueredo J-M García-Ael C Gragnano A , et al. Well-being at work after return to work (RTW): a systematic review. Int J Env Res Pub Health 2020; 17: 7490. 33076302 10.3390/ijerph17207490 PMC7602369

[32] Lydell M Hildingh C Soderbom A , et al. Future challenges for occupational health services can be prevented by proactive collaboration with the companies using the services: a participatory and reflection project. J Multidiscip Healthc 2017; 10: 217–225. 28579793 10.2147/JMDH.S131382 PMC5449106

[33] Vogt K Hakanen JJ Jenny GJ , et al. Sense of coherence and the motivational process of the job-demands–resources model. J Occup Health Psychol 2016; 21: 194. 26690920 10.1037/a0039899

[34] Lydell M Hildingh C Söderbom A , et al. How to promote workplace health in order to work into old age: Experiences from employees in an industrial setting. Scientifica 2019; 2019: 1–8. 10.1155/2019/3942569 PMC6466871 31065397

[35] Ejlertsson L Heijbel B Andersson IH , et al. Strengthened workplace relationships facilitate recovery at work–qualitative experiences of an intervention among employees in primary health care. BMC Fam Pract 2021; 22: 1–10. 33750316 10.1186/s12875-021-01388-x PMC7942012

[36] Bendixen L Scheel T . The long-term effects of job demands on psychological detachment and health: the moderating role of leader behaviour. Work & Stress 2023; 38: 1–20.

[37] Neidlinger SM Felfe J Schübbe K . Should I stay or should I go (to the office)?—effects of working from home, autonomy, and core self–evaluations on leader health and work–life balance. Int J Env Res Pub Health 2022; 20: 6. 36612327 10.3390/ijerph20010006 PMC9819704

[38] Martin L Pénard T Poussing N . Are employees happier when staying connected with their companies outside working hours? Soc Sci Comput Rev 2022; 40: 1035–1053.

[39] Antonovsky A . Unraveling the mystery of health: How people manage stress and stay well. San Francisco: Jossey-bass, 1987.

[40] Løseth GE Eikemo M Trøstheim M , et al. Stress recovery with social support: a dyadic stress and support task. Psychoneuroendocrinology 2022; 146: 105949. 36240542 10.1016/j.psyneuen.2022.105949

[41] Antonovsky A . Health, stress, and coping. New perspectives on mental and physical well-being. San Francisco: Jossey-Bass, 1979, pp.12–37.

[42] Pasanen J Luoma A . How can social insurers promote return to work in occupational rehabilitation? A quantitative, cross-sectional study. BMC Pub Health 2021; 21: 1–11. 34530777 10.1186/s12889-021-11758-w PMC8444422

[43] Nappo N . Job stress and interpersonal relationships cross country evidence from the EU15: a correlation analysis. BMC Pub Health 2020; 20: 1–11. 32689996 10.1186/s12889-020-09253-9 PMC7372642

[44] Ejlertsson L Heijbel B Ejlertsson G , et al. Recovery, work-life balance and work experiences important to self-rated health: a questionnaire study on salutogenic work factors among Swedish primary health care employees. Work 2018; 59: 155–163. 29439377 10.3233/WOR-172659 PMC5817904

[45] Wagman P Hakansson C Jacobsson C , et al. What is considered important for life balance? Similarities and differences among some working adults. Scand J Occup Ther 2012; 19: 377–384. 22250769 10.3109/11038128.2011.645552

[46] Karlsson L Ivarsson A Erlandsson LK . Exploring risk factors for developing occupational ill health - departing from an occupational perspective. Scand J Occup Ther 2021; 38: 1–10. 10.1080/11038128.2021.1936160 34184961

[47] Lindgren B-M Lundman B Graneheim UH . Abstraction and interpretation during the qualitative content analysis process. International Journal of Nursing Studies 2020; 108: 103632. 32505813 10.1016/j.ijnurstu.2020.103632

[48] Bengtsson M . How to plan and perform a qualitative study using content analysis. Nurs Plus Open 2016; 2: 8–14.

[49] Graneheim UH Lindgren B-M Lundman B . Methodological challenges in qualitative content analysis: a discussion paper. Nurse Educ Today 2017; 56: 29–34. 28651100 10.1016/j.nedt.2017.06.002

[50] Kvale S Brinkmann S . Interviews: Learning the craft of qualitative research interviewing: sage. 2009.

[51] Elo S Kääriäinen M Kanste O , et al. Qualitative content analysis: a focus on trustworthiness. SAGE Open 2014; 4: 2158244014522633.

